# Advancement in Multi-omics approaches for Uterine Sarcoma

**DOI:** 10.1186/s40364-024-00673-y

**Published:** 2024-10-29

**Authors:** Wuyang Wang, Yu Hu, Fangfang Fu, Wu Ren, Tian Wang, Shixuan Wang, Yan Li

**Affiliations:** Department of Obstetrics and Gynecology, Tongji Hospital, Tongji Medical College, Huazhong University of Science and Technology, 1095 Jiefang Anv. Wuhan, Wuhan, Hubei 430030 P.R. China

**Keywords:** Uterine Sarcoma, Multi-omics, Biomarker Discovery, Diagnosis, Prognosis, Treatment

## Abstract

Uterine sarcoma (US) is a rare malignant tumor that has various pathological types and high heterogeneity in the female reproductive system. Its subtle early symptoms, frequent recurrence, and resistance to radiation and chemotherapy make the prognosis for US patients very poor. Therefore, understanding the molecular mechanisms underlying tumorigenesis and progression is essential for an accurate diagnosis and targeted therapy to improve patient outcomes. Recent advancements in high-throughput molecular sequencing have allowed for a deeper understanding of diseases through multi-omics technologies. In this review, the latest progress and future potential of multi-omics technologies in US research is examined, and their roles in biomarker discovery and their application in the precise diagnosis and treatment of US are highlighted.

## Background

 Uterine sarcoma (US) is an uncommon tumor found in the female reproductive system and comprises approximately 1% of all malignancies in the female genital tract and 3–7% of tumors found in the uterus. Owing to the rarity of US and lack of specific biomarkers, there is no reliable preoperative diagnostic method for US. Imaging methods such as preoperative ultrasound, CT, and PET-CT have limitations with regard to distinguishing between benign uterine leiomyoma (ULM) and malignant US. Recently, diffusion weighted imaging(DWI) has been suggested as a potential diagnostic tool, however, further validation of its accuracy is still needed [1, 2]. Studies have shown that 0.5% of patients who receive surgical treatment for uterine leiomyoma are diagnosed with US by means of post-surgical histopathological examination [3]. Patients with unidentified US before surgery may experience accidental “peritoneal seeding of the tumor”, leading to diffuse dissemination of malignant tissue in the abdominal cavity and adverse effects on prognosis. In addition, US has a high recurrence rate ranging from 53 to 71% [4]. Due to the rarity and heterogeneity of US, the optimal treatment method remains controversial. Currently, the primary treatment is surgery, and radiotherapy and chemotherapy may serve as adjunctive treatments and palliative treatments for patients with metastasis or recurrence. Compared with other malignant tumors, US tends to result in early metastasis and recurrence; therefore, the overall prognosis is poor, with a 5-year survival rate of 15–25% and a median survival time of 10 months [1, 5]. Clinicians face urgent challenges in improving diagnostic accuracy and enhancing the effectiveness of treatment for US.

With the advent of the “omics” concept [6] and advancements in next-generation sequencing (NGS) technology, high-resolution mass spectrometry, and integrated multi-omics analysis methods and databases, omics technology has transitioned from traditional omics to multi-omics technology. Powered by multi-omics, systems biology research is paving the way for a new era in life science research. Research institutions and scholars are now collecting samples from patients to evaluate them at genomic, epigenomic, transcriptomic, proteomic, and metabolomic levels and to obtain disease-specific omics information through microarrays or high-throughput testing. This innovative approach has the potential to transform our understanding of diseases and their underlying mechanisms, potentially leading to more effective and personalized treatment strategies. By leveraging computer technology, researchers are able to analyze pathogenic genes and key molecular pathways associated with a specific disease, and identify molecular markers customized for its diagnosis and treatment [7]. This precision approach allows for a more accurate and targeted comprehension of the disease, potentially resulting in enhanced outcomes for patients.

To address the challenges of the clinical diagnosis and treatment of US, researchers are actively utilizing multi-omics approaches to study this malignancy. Their focus is on mapping the molecular landscape of US via advanced high-throughput technologies and establishing molecular classifications to improve diagnostic accuracy and prognostic prediction. Over the past few decades, omics applications in US have led to a deeper understanding of disease development and pathogenic mechanisms. In this review, we attempt to summarize the recent advances of multi-omics technology in the diagnosis and treatment of US and discuss the current challenges as well as perspectives for the near future.

## Clinical pathology and molecular pathology of uterine sarcoma

**Fig. 1:**
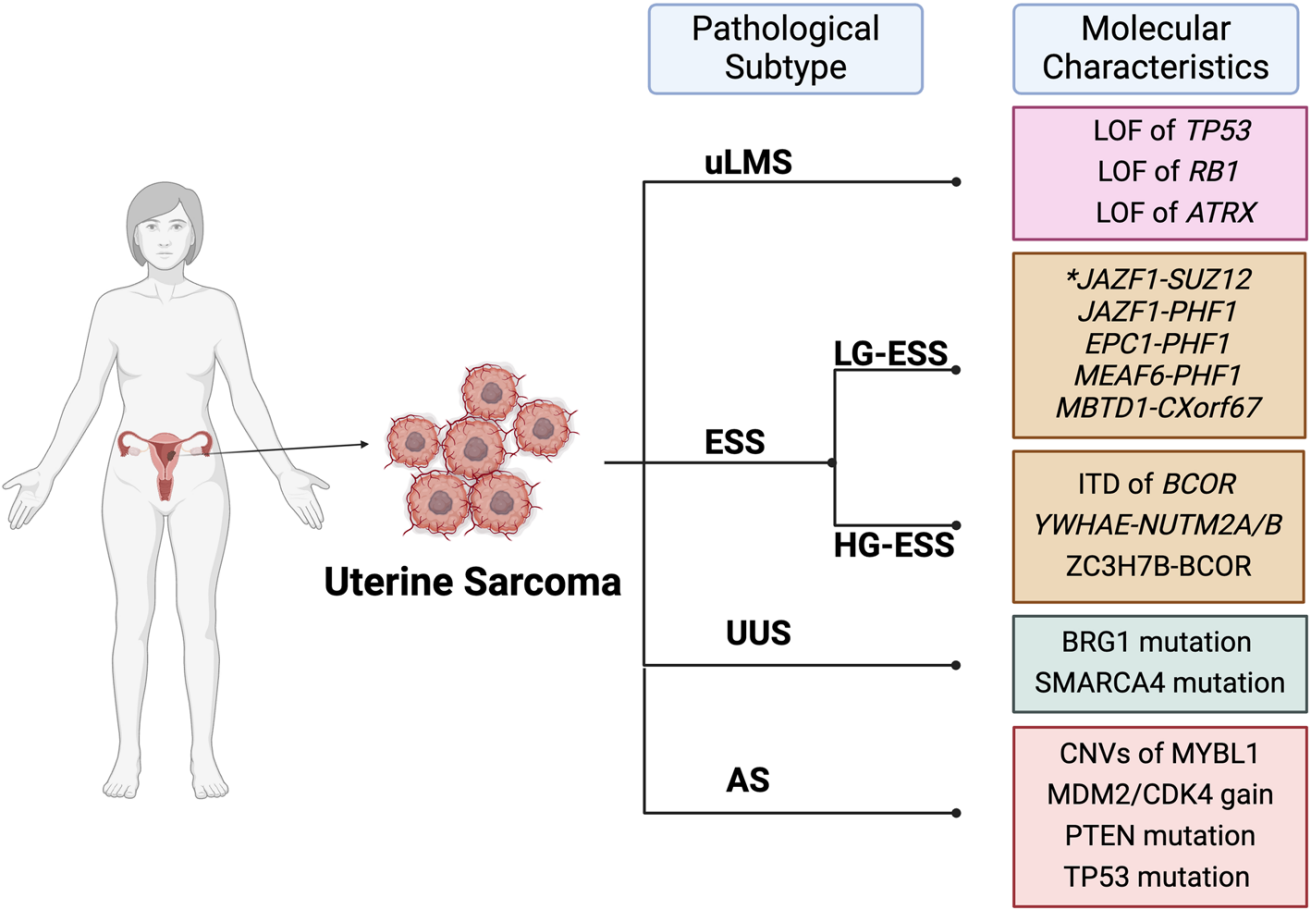
Molecular characteristics of different pathological types of uterine sarcoma. US can be roughly categorized in to four types in histopathology, such as uterine leiomyosarcoma (uLMS), endometrial stromal sarcoma (ESS), undifferentiated uterine sarcoma (UUS) and other rare types such as adenosarcoma (AS). ESS can be categorized into two distinct subtypes: low-grade endometrial stromal sarcoma (LG-ESS), high-grade endometrial stromal sarcoma (HG-ESS). Utilizing NGS and other genetic technologies, these subtypes have been identified to possess molecular characteristics. (Created with BioRender.com)

US is a heterogeneous malignant tumor that originates from uterine stromal tissue, such as the endometrial stroma, uterine muscle layer, and connective tissue [8]. According to the 2014 cancer report of the International Federation of Gynecology and Obstetrics (FIGO) [1], US can be categorized into four types according to histopathology: uterine leiomyosarcoma (uLMS), endometrial stromal sarcoma (ESS), undifferentiated uterine sarcoma (UUS) and other rare types such as adenosarcoma (AS). US is a heterogeneous tumor that displays significant molecular diversity even within a single histological subtype. (Fig. 1) Sarcomas of distinct molecular subtypes exhibit varying degrees of aggressiveness. Recognizing this, the National Comprehensive Cancer Network (NCCN) has integrated molecular profiling information for US into its guidelines to increase the accuracy of the diagnosis, classification, and treatment of these malignancies [9, 10].

### Uterine Leiomyosarcoma

Initially, uLMS was the most prevalent subtype of US, accounting for approximately 63% of all USs. These tumors arise either directly from smooth muscle cells or from mesenchymal stem cells that differentiate into smooth muscle cells [1]. In accordance with the National Comprehensive Cancer Network(NCCN) guidelines for uterine neoplasms, uLMS also includes epithelioid and myxoid variants. Predisposing factors for uLMS include LiFraumeni syndrome, hereditary retinoblastoma, and radiation exposure [11]. In histopathology, uLMS exhibits polymorphic differentiated cells of smooth muscle, displaying nuclear atypia and a high mitotic rate, typically exceeding 15 mitoses per 10 high-power fields (MF/10HPF). Immunohistochemically, despite the morphological differences among subtypes, they all express varying degrees of smooth muscle markers such as desmin, smooth muscle actin (SMA), and caldesmon. Additionally, uLMS often exhibits immunoreactivity to CD10 and epithelial markers including keratin and EMA [12]. The high level of Ki67 in uLMS, along with the overexpression of p16, are promising adjunct immunomarkers that may aid in distinguishing between benign and malignant uterine smooth muscle tumors [13]. 

At the molecular biology level, the Cancer Genome Atlas Research Network (TCGA) integrated multi-omics data from 206 adult soft tissue sarcomas, including 27 uLMS cases, to delineate the multiplatform molecular landscape. Their findings revealed that sarcomas are characterized primarily by copy number variations (CNVs), coupled with low mutational loads and only a handful of genes (TP53, ATRX, RB1) that are recurrently mutated across various sarcoma types [14]. Consistent with this, another study on uLMS reported similar results, including loss-of-function mutations or homozygous deletions of TP53, RB1, and ATRX. Notably, uLMS presented the highest rate of PDCD1 homozygous deletions across all the tumors examined [15]. Additionally, mutations in the MED12 gene were detected in nearly 70% of malignant uLMS cases [16]. Furthermore, advancements have been made in understanding the molecular variants associated with uLMS. For example, PGR fusion has been identified as a biomarker for epithelioid leiomyosarcoma in a subset of cases with uniform nuclear atypia and rhabdoid features [17]. On the other hand, the expression of PLAG and/or the PLAG1 fusion and/or Desmin serves as a confirmatory marker for myxoid leiomyosarcoma [18]. Regarding the prognostic application of these biomarkers, p53, p16, Ki67, and Bcl-2, which are crucial ancillary parameters, have been explored in uLMS to predict patient outcomes [13]. Overall, uLMS is genetically unstable, exhibiting complex structural chromosomal abnormalities and highly disrupted gene regulation, potentially reflecting the cumulative effect of multiple genetic defects [1]. 

### Endometrial Stromal Sarcoma

Endometrial stromal sarcoma is a rare subtype, comprising approximately 21% of all uterine sarcomas. Pursuant to the 2014 World Health Organization classification (WHO), this malignancy can be categorized into two distinct subtypes: low-grade endometrial stromal sarcoma (LG-ESS) and high-grade endometrial stromal sarcoma (HG-ESS) [19]. With the use of NGS and other genetic technologies, these subtypes have been observed to have distinct morphologic and molecular characteristics (Table 1). Consequently, the prognosis of each subtype of endometrial stromal sarcoma (ESS) varies significantly. LG-ESS, characterized as an indolent tumor, is associated with favorable long-term survival outcomes. Conversely, HG-ESS is highly aggressive and is often associated with a poor prognosis. On histological examination, LG-ESS comprises tumor cells resembling proliferative endometrial stromal cells. These tumor cells demonstrate diffuse infiltrative growth and cytologically bland spindle cell proliferation, akin to proliferative endometrial stroma. Additionally, the presence of myoinvasion and/or lymphatic vascular invasion (LVSI) serves as the pathological basis for diagnosing LG-ESS [1, 9]. The results of molecular pathological examination revealed that: the most common cytogenetic abnormality is a translocation involving chromosomes 7 and 17 [ t( 7 ; 17 ) ( p15 ; q21) ] for LG-ESS, which is a JAZF1-SUZ12 gene fusion [20]. HG-ESS has features that lie somewhere between LG-ESS and UUS, indicating an intermediate nature among these subtypes [21]. The tumors were composed of uniformly high-grade round or spindle cells exhibiting active mitotic figures, occasionally displaying components characteristic of LG-ESS. These tumors exhibit an expansive, penetrating, and invasive growth pattern [22]. While distinct molecular subtypes exhibit varying histologic features, molecular pathological examination revealed that HGESS undergoes specific molecular genetic alterations. These include the rearrangement of the YWHAE-FAM22 A/B gene [23], the ZC3H7B-BCOR fusion gene, and internal tandem duplication (ITD) of the BCOR gene [24]. With respect to HG-ESS, several mutated genes have been reported: YWHAE, NUTM2, EPC1, SUZ12, BCOR, BRD8, PHF1, ZC3H7B, TPR, NTRK1, LMNA, TPM3, RBPMS, NTRK3, EML4, COL1A1, PDGFB, and STRN [21]. 

**Table 1:** Molecular features to define different subtypes endometrial stromal sarcoma

ESS subtypes	Gene fusion	Detection methods	Ref
HG-ESS	YWHAE-NUTM2A/B fusion ZC3H7B-BCOR fusion BCOR ITD	Targeted RNA-seq	[24]
HG-ESS	BCORL1 rearrangements BCORL1 mutations homozygous BCORL1 deletion	DNA-seq and RNA-seq	[25]
HGESS-BCOR	MDM2 amplification	Gene array	[26]
ESS (close to LG-ESS)	KAT6B::KANSL1 Fusion	bulk RNA-seq CGH	[27]
LG-ESS	MBTD1-CXorf67 fusion	bulk RNA-seq	[28]
LG-ESS	JAZF1-PHF1 fusion EPC1-PHF1 fusion	RACE-PCR	[29]
LG-ESS	BRD8-PHF1 fusion	RNA-seq	[30]
LG-ESS	EPC2-PHF1 fusion	RNA-seq	[31]
LG-ESS	JAZF1-SUZ12 fusion	RNA-seq	[32]

*ITD* Internal tandem duplication, *CGH* array comparative genomic hybridization, *FISH* fluorescence in situ hybridization, *IHC* Immunohistochemistry, *RACE-PCR* 3 V-Rapid amplification of cDNA ends PCR, *MPS* Massively parallel sequencing

### Undifferentiated uterine sarcoma

Undifferentiated uterine sarcoma (UUS) refers to highly aggressive mesenchymal tumors lacking specific differentiation. Tumor cells exhibit a high degree of nuclear pleomorphism, active mitotic activity, and destructive invasion into the myometrium [33]. The characteristics of morphology and immune staining are the lack of any evidence of cell differentiation and its diagnosis is often exclusive [34]. With the development of sequencing technology, some UUSs have been reclassified as other histological subtypes of uterine sarcomas by means of their molecular markers [35]. For example, USs with NTRK alterations which are classified as UUSs have been reclassified as HG-ESS [36]. BRG1 loss and/or SMARCA4 mutation have identified as diagnostic biomarkers [37]. 

### Adenosarcoma

Adenosarcoma is a malignant tumor composed of benign epithelial components and malignant mesenchymal components [10]. Pathologically, the tumor is lobulated, with fissures or dilated glandular components lined with benign endometrial epithelium, and neoplastic stromal cells surrounding the glands, which are abundant and of varying degrees. Atypia and mitotic figures are generally rare or absent. In most cases, the sarcoma components in AS are homologous, resulting in endometrial stromal or smooth muscle differentiation [33]. When sarcoma constitutes 25% or more of the tumor volume, indicating a highly aggressive tumor, the prognosis is unfavorable. The possible biomarkers of AS that have not received ancillary testing include: 8q13 amplification and CNVs of MYBL1; NCOA2/3 fusions; and rare FGFR2, KMT2C, DICER1, MDM2/CDK4 and TERT amplifications [9, 38]. 

## Multi-omics in Uterine Sarcoma

### Sample source and Novel Detected Approach

For a benign or malignant tumor diagnosis, pathologic conclusion may be the best way to judge. Surgical specimens obtained by curettage of the uterine cavity are the main sources of tumor tissue. Solid tumor biopsy is performed by obtaining tumor tissue after surgery for pathological testing, IHC or NGS testing. Fresh frozen (FF) / Formalin-fixed paraffin embedding (FFPE) samples obtained from solid tumor biopsies are also widely used in scientific research. Moreover, with the proposal of the concept of “precision medicine” and the demand for personalized treatment, liquid biopsy (LB), a quick and easy test for cancer, has become a promising method for tumor diagnosis [39]. In contrast to solid tumor biopsy, LB is a noninvasive method, that can used analyze molecular biomarkers sampled from body fluids such as blood [40] and pleural effusion [41]. Circulating free DNA(cfDNA), circulating tumor DNA(ctDNA), exosomes, circulating RNA, mRNA, microRNA, long noncoding RNA(lnc-RNA), proteins, peptides, and metabolites can be isolated from these samples [42]. These samples can be used to monitor the tumor cells status and analyze the molecular subtypes of tumors [43]. This approach is highly compatible with genetic technologies, contributing to the association between LB and multi-omics.

Currently, the analysis of cell-free DNA (cfDNA) and circulating tumor DNA (ctDNA) has emerged as a complementary method to tissue-based genomic testing for US. The application of genetic technologies to detect and measure copy number variations (CNVs) in cfDNA/ctDNA has been thoroughly assessed in numerous cancer types, providing valuable insights into disease progression and patient outcomes [44, 45]. **(**Table 2**)** Detection of ctDNA has become a new method for identifying cancer-causing mutations, measuring disease burden, performing clinical predictions, and evaluating tumor treatment response [46]. Monitoring ctDNA dynamics is expected to detect disease progression in various malignancies in advance [47]. However, there are currently certain limitations in the field of LB combined with US. The concentration of cfDNA/ctDNA in blood is quite low ranging from 10 to 100 ng/ml [48], and more precise detection methods are needed.

**Table 2:** Studies in cfDNA/ctDNA of Uterine Sarcoma

US subtype	Tissue resource	Detected thing	Methods	Conclusion	Ref
uLMS	serum	LMW cfDNA	DNA chip	Serum LMW cfDNA concentration is associated with overall survival	[49]
uLMS	plasm	cfDNA	ULP-WGS	Higher levels of ctDNA correlate with tumor size and disease progression.	[50]
uLMS/extra-LMS	plasm	ctDNA	ctDNA arrays	ctDNA levels tracked longitudinally with progression of disease and response to therapy	[51]
uLMS	plasm	cfDNA	WGS Exome-seq	Propose that the detection of CNAs or point mutations in selected tumor suppressor genes in ctDNA may favor a diagnosis of LMS and identified two recurrently mutated genes in LM tumors (MED12 and ACLY).	[52]

*LMW cfDNA* Low molecular weight cfDNA, *ULP-WGS* ultra-low passage whole-genome sequencing

Although LB has many advantages in terms of diagnosis and prognosis, such as being noninvasive, data-multidimensional, simplicitye and potential for use in tumor precision medicine, solid tumor biopsy still plays a major role in diagnosis and treatment.

### Genomics

Genomics, the discipline that explores the genome and leverages the genetic material of organisms, has been revolutionized by technologies such as NGS. These advanced methods, including whole genome sequencing (WGS) and whole exome sequencing (WES), enable us to comprehensively map the genetic landscape of diseases and decipher the intricate links between diseases and their underlying genetic components [53]. Genomics technology is widely used to detect single nucleotide variations (SNVs), copy number variations (CNVs), and chromosomal rearrangements, providing invaluable insights into the genetic basis of various conditions [54]. The stability of DNA allows a wide range of sample sources for genome sequencing, which, to a certain extent, has not hindered the genome research of rare tumors. In the field of US, there have several investigations of the genomic profiles of various US subtypes and of potential molecular markers. Through the tireless efforts of researchers, the genomic landscape of US is slowly being pieced together with greater precision.

#### Genomics in Molecular mechanisms and Biomarker Discovery

Through genomic profiling, several specific molecular features of US have been discovered. The TCGA database revealed that sarcomas are characterized by a higher frequency of CNVs and relatively low somatic tumor mutation burdens (TMB) than other types of tumors. Detailed analysis of the genomic data revealed three genes that are significantly mutated in uLMS: TP53, ATRX, and RB1. Notably, the TP53 gene, which plays a pivotal role as a tumor suppressor, is mutated in many solid tumors. These mutations can lead to uncontrolled cell proliferation without proper division, serving as a fundamental molecular mechanism underlying the development of most tumors. Furthermore, TP53 mutations are particularly prevalent in leiomyosarcomas, highlighting the importance of this gene in the pathogenesis of this subtype of sarcoma [14]. Furthermore, research conducted by Martee L. Hensley et al. revealed that the predominant mutations were loss-of-function alterations in TP53 (56%), RB1 (51%), and ATRX (31%). They described the genomic profile of US through the utilization of MSK-IMPACT (a high-throughput sequencing method). Additionally, homozygous deletions of BRCA2 were observed in 5% of the patients, and a sustained partial response was noted in patients with uLMS harboring somatic BRCA2 mutations, when treated with PARP-inhibitor therapy. Moreover, comparative analysis of primary and metastatic tumors revealed that metastases had a higher frequency of PTEN alterations than did primary tumors [55]. In another study aimed at discovering driver genes in uLMS to identify driver mutations and potential therapeutic targets, researchers performed WES on the specimens of three patients with uLMS. Their analysis revealed four genes harboring somatic SNVs that could serve as driver mutations. Notably, SHARPIN was amplified in two patients and mutated in the other patients. Additionally, the knockdown of SHARPIN expression in US cell lines decreased cell growth and colony formation. These findings suggest that SHARPIN may serve as a potential therapeutic target because of its association with the diagnosis of uLMS [56]. Transposons, which are mobile genetic elements, play an important role in shaping our genomes [57, 58]. Transposon-insertion sequencing enables a direct correlation between phenotype and genotype, ultimately facilitating the assignment of gene function [59]. Using this technology, Michiko Kodama et al. used the sleeping beauty transposon to develop a mouse model for studying tumor progression. Analysis of omics data obtained through transposon insertion sequencing of sarcomas pinpointed ZNF217 as a potential oncogenic driver and therapeutic target, whereas NRDC emerged as a metastasis-specific driver [60]. Genetic technologies have revealed certain biomarkers that may delineate a novel molecular subtype. In a particular study, researchers discovered a case of HGESS-BCOR fusion associated with the ZC3H7B-BCOR gene, which exhibited amplification of the MDM2 locus. This amplification was confirmed through array-based copy number analysis and fluorescence in situ hybridization (FISH) analysis of the tumor tissue. Furthermore, immunohistochemical (IHC) analysis validated MDM2 expression in four additional HGESS-BCOR cases. These findings indicate that HGESS-BCOR carries MDM2 amplification, providing valuable insights into the molecular characteristics of this subtype and potential therapeutic targets [26]. BCORL1, a transcriptional corepressor homologous to BCOR, has been extensively studied. In a recent investigation, 12 USs harboring BCORL1 mutations were carefully selected for analysis. Among these samples, five exhibited a rearrangement in BCORL1, specifically involving JAZF1-BCORL1, EP300-BCORL1, or an internal rearrangement within the gene. Another five samples displayed loss-of-function mutations in BCORL1, characterized by specific mutations such as T513fs22, P600fs1, R945*, R1196*, or R1265fs*4. These mutations were identified through meticulous analysis of NGS data. Furthermore, two samples revealed a homozygous deletion of BCORL1. When these samples were retyped, it became evident that they received a different diagnosis than that assigned prior to genomic testing. This finding underscores the importance of genomic profiling in accurately diagnosing and understanding the molecular basis of US [25]. The sequencing of the queue reveals the somatic mutation of US only to a certain extent and provides a specific hypothetical basis for the molecular mechanism of tumorigenesis and development. However, compared with that in other tumor origin studies, the progress in US research is not significant. For example, LCM-WES (laser capture microdissection-whole exome sequencing) can used to conduct multisite sequencing of acral melanoma to reveal the clonal evolution pattern of tumor progression [61]. 

Like other tumors, different histological subtypes of US can be divided into different molecular subtypes on the basis of genomic signatures. As one type of genomic signature, biomarkers discovered through genomics methods play a crucial role in precision diagnosis. Some biomarkers or potential biomarkers have been detected via genomic sequencing methods (Table 3). First, distinguishing between US and usual-type leiomyomas has been a difficult for clinicians. WGS between uLMS and several benign variants have revealed some oncogenic alterations [62] and research has revealed that the loss of the dystrophin gene is common in dedifferentiated leiomyosarcomas of gynecologic origin with poor outcomes [63]. baBrian Vadasz et al. conducted a study encompassing a range of leiomyoma subtypes, including leiomyomas with bizarre nuclei (LM-BNs) and fumarate hydratase-deficient leiomyomas (FH-LMs). Their findings also revealed that dystrophin was detectable in a high proportion of leiomyomas, whereas its presence was significantly lower in leiomyosarcomas, detected in only 18% of cases. To further explore the potential mechanism underlying the loss of dystrophin expression in leiomyosarcomas and LM-BN, researchers have conducted WGS on LM-BN cases and leiomyosarcomas. Their analysis of CNVs within the dystrophin genomic region revealed that all 10 uLMS cases exhibited CNVs loss in the dystrophin gene. In contrast, this loss was observed in only 33% (5/15) of the LM-BN cases. These findings offer valuable insights into the molecular pathways that govern various forms of ULM and uLMS, aiding in more accurate detection and potential treatment options [64]. Some molecular characteristics of ESS have been described in the previous text, but large-scale sequencing studies have not yet revealed the molecular characteristics of other rare types of US.

Genome sequencing is used to find high-frequency mutated genes or germline pathogenic genes in diseases to achieve the goal of precision medicine. This method is more common in the diagnosis of genetic diseases. Additionally, in the field of cancer, genetic diagnosis is challenging compared with traditional methods. For example, the NCCN guidelines recommend TCGA molecular typing diagnosis for patients with endometrial cancer [65]. However, owing to the scarcity of samples, the lack of validation of the discovered biomarkers of US in large-scale cohorts has hindered their application in clinical diagnosis.

**Table 3:** Biomarkers discovered to diagnosis and distinguish US by genomics

US subtype/number	Tissue source	Detection Methods	Biomarker	Ref
84 uLMS	FF/FFPE	WGS, RNA-seq, SNP arrays	VIPR2	[15]
34 uLMS	FFPE	WGS	dystrophin	[63]
uLMS	uLMS cell line FFPE	Transposon seq	ZNF217 NRDC	[60]
13 uLMS	FF	WES, TP53 sequencing	SHARPIN	[56]
36HG-ESS	FF	CNV arrays	CDKN2A	[66]
STUMP	FFPE	CGH	BCL2	[67]

*STUMPs* smooth muscle tumors of uncertain malignant potential

#### Genomics in Clinical Therapy and Prognosis

To investigate the mechanisms underlying the expression of tumor driver genes in US and their potential drug resistance, researchers plan to observe the mutation status of US genes under external drug treatment. By employing high-throughput sequencing in US samples that exhibit sensitivity to a specific test drug, researchers aim to analyze the genomic data obtained, thereby enhancing targeted therapy strategies and assessing the effectiveness of immunotherapy.

According to genomic database analysis, uLMS frequently exhibits deficiencies in DNA repair mechanisms, especially in the pathway for homologous recombination repair (HRR) [14, 68]. One study employed a 16-gene panel NGS approach to investigate the prognostic differences between primary and recurrent tumors, focusing primarily on HRR genes. The research also highlighted that patients harboring somatic mutations in BRCA1/2 might potentially benefit from PARP inhibitors, on the basis of the frequency of BRCA-related mutations, albeit with a limited sample size [69]. Researchers noted that PARP inhibitors had long-lasting clinical advantages in four patients with uLMS who presented with BRCA2 deficiency, suggesting a promising treatment strategy for this particular group of individuals. Additionally, PARP-inhibitors demonstrated durable clinical benefits in uLMS patients with BRCA2 inactivation [70]. Temozolomide with PARP inhibitors, such as olaparib, has shown promising results in prospective clinical trials for patients with uLMS. A study involving 22 uLMS patients revealed that the combination of olaparib and temozolomide resulted in an objective response rate of 27% and a median progression-free survival (PFS) time of 6.9 months. These findings suggest that this treatment approach may be effective in managing uLMS and improving patient outcomes. Further research and larger clinical trials are warranted to confirm these results and assess the long-term benefits and potential side effects of this combination therapy [71]. Poly(ADP-ribose) polymerase 1 (PARP-1) specific inhibitors and DNA polymerase theta (Pol-theta) inhibitors could also be valuable for research on uLMS. ATX-101, a cell-penetrating peptide, has shown promise as a new therapeutic agent. It effectively disrupts the interaction between proliferating cell nuclear antigen (PCNA), a key scaffolding protein, and essential proteins responsible for the DNA damage response and intracellular signaling pathways. Currently, a phase II clinical trial is underway to evaluate the efficacy of ATX-101 as a monotherapy in patients with leiomyosarcoma. (NCT05116683)

On the basis of the results of the gene enrichment analysis, the PI3K/mTOR/AKT signaling pathway is activated in uLMS via different mechanisms, including the amplification of mTOR, RICTOR, AKT, or insulin-like growth factor 1, as well as the loss of tumor suppressors such as PTEN [14, 55]. Prior studies have reported an association between PTEN loss and resistance to anti-PD-1 checkpoint blockade therapy in patients with metastatic uLMS. Suzanne George et al. described a case of metastatic uLMS in a patient who did not receive any therapy following initial surgery. During anti-PD-1 monotherapy, the patient developed a drug-resistant metastasis. Following a second resection, WES and bulk RNA-seq were conducted on both the primary tumor and the metastatic tumor. Gene profiling revealed that biallelic PTEN loss was associated with the induction of an immunosuppressive microenvironment. These findings provide crucial insights into the molecular mechanisms underlying drug resistance in US, offering potential targets for future therapeutic strategies [72]. Monotherapy with mTOR inhibitors has demonstrated limited efficacy. For example, in the phase II trial investigating the dual mTORC1/2 inhibitor sapanisertib, the objective response rate for patients with leiomyosarcoma was merely 3%, and the median PFS was only just 2.1 months [73]. A phase II trial was conducted to evaluate the efficacy of the CDK4/6 inhibitor ribociclib in combination with the mTOR inhibitor everolimus for patients with LMS, which retains retaining Rb expression, among which 58% had uLMS. The study aimed to consider the combination promising if at least 8 out of 24 patients remained progression-free at 16 weeks. However, the therapy succeeded in meeting this criterion in only 6 out of 24 patients (25%), with a median PFS of 14 weeks [74]. 

Mutations that activate the PI3K /Akt signaling pathway and inactivate the TP53 tumor-suppressor gene are common and essential mechanisms of cancer cell proliferation and escape from preprogrammed cell death [75]. Yu Xia et al. carefully selected four tumors that had undergone treatment with rAd-p53 (Gendicine^®^) and confirmed the effectiveness of rAd-p53 in US. In their analysis, they discovered a total of 30 mutated genes. Notably, TP53 mutations were detected in two of the patients. Additionally, in the remaining two patients, they identified mutated genes such as CREBBP, LYN, CDKN2A, and JAK2, which are located upstream of the TP53 gene and have a significant impact on its function. These findings strongly suggest that the TP53 signaling pathway plays a pivotal role in the tumorigenesis of US, similar to its role in other types of tumors. These results suggest that patients harboring TP53 mutations might benefit from treatment with rAd-p53 [76]. Recent preclinical studies indicate that CHK1, WEE1, and ATR inhibitors could be active in uLMS. However, it is essential to conduct further research to prioritize the clinical assessment of these agents [63]. 

### Epigenomics

**Fig. 2:**
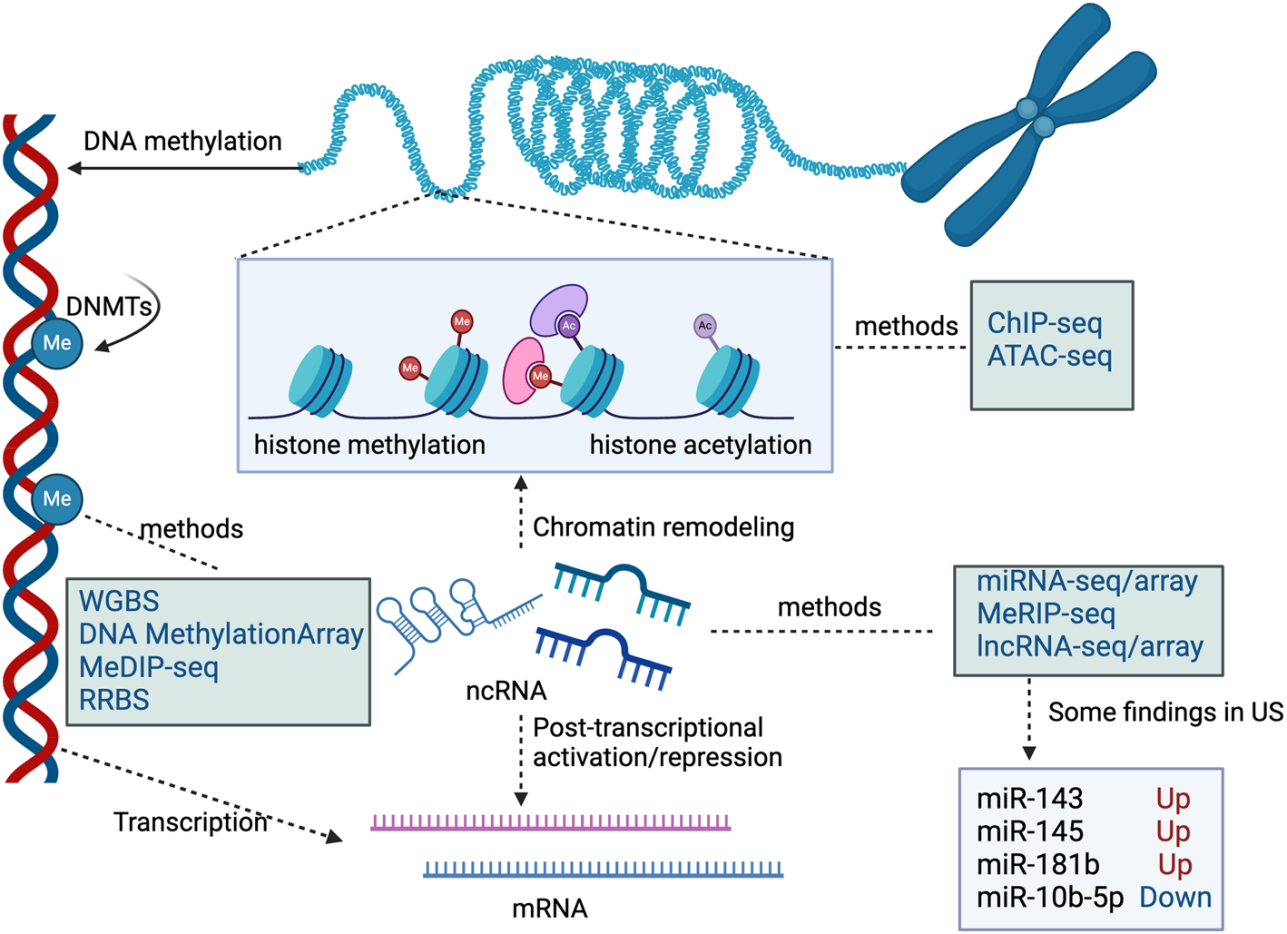
Epigenetic regulation mechanisms and corresponding molecular biology sequencing methods. Epigenetic modifications primarily encompass DNA methylation, histone modifications, and alterations in ncRNA(non-coding RNAs). Corresponding omics approaches have been developed to study these changes. At the chromatin level, sequencing methods such as ChIP-seq (Chromatin Immunoprecipitation followed by high-throughput sequencing) and ATAC-seq (Assay for Transposase-Accessible Chromatin with high throughput sequencing) are utilized. For DNA-level modifications, there are sequencing methods like WGBS(Whole Genome Bisulfite Sequencing)，RRBS(Reduced Representation Bisulfite Sequencing) and MeDIP-seq(Methylated DNA Immunoprecipitation Sequencing). On the RNA level, miRNA-seq, lncRNA-seq and MeRIP-seq(Methylated RNA Immunoprecipitation Sequencing) are employed. These methodologies collectively contribute to uncovering the epigenetic mechanisms underlying uterine sarcomas. For example, through miRNA-seq, researchers found that the expressions of miR-143, miR-145b, and mi-181b were downregulated, whereas the expression of miR-10b-5p was upregulated. DNMTs(DNA Methyltransferases) (Created from BioRender.com)

Epigenetic changes represent a key mechanism underlying tumorigenesis [77]. Epigenetic modifications primarily include DNA methylation, histone modifications, and alterations in noncoding RNAs [78]. The field of epigenetics is constantly witnessing the emergence of many high-throughput methodologies that enable the interrogation of intricate epigenetic changes. (Fig. 2) These advancements have significantly broadened our comprehension of the intricate mechanisms underlying gene regulation and gene expression.

At the level of chromatin modification, due to the limitation of clinical samples of uterine sarcoma, no researchers have conducted sequencing studies on US through ChIP-seq(Chromatin Immunoprecipitation followed by high-throughput sequencing) or ATAC-seq(Assay for Transposase-Accessible Chromatin with high throughput sequencing). While, researchers found FASN reprograms the uLMS epigenome through chromatin remodeling to promote the malignant phenotype through ChIP-seq in SK-UT-1 cell lines [79]. In particular, epigenetic alterations caused by DNA methylation have been implicated in US studies. TCGA has proven that the methylation pattern of uLMS is different from that of other soft tissue sarcomas [14], and identifying the methylation profiles of different histological subtypes of US is highly important for precision diagnosis. Researchers have conducted array-based DNA methylation analysis on a diverse set of uterine tumors, encompassing uterine leiomyomas, uLMSs, uterine tumors resembling ovarian sex cord tumors (UTROSCTs), LG-ESSs, and HG-ESSs. The resulting data comprehensively characterized the methylation profile of these gynecologic tumors, providing a deeper understanding of the epigenetic landscape in these malignancies and demonstrating significant potential in diagnosis [80]. Smooth muscle tumors of uncertain malignant potential (STUMPs) are a type of tumor with uncertain malignant potential, and studying epigenetic changes is valuable for understanding the occurrence and development of tumors and for predicting their prognosis. A DNA methylation array analysis was conducted on uLMS and STUMPs. Notably, a general hypomethylation of CpG islands (CGIs) was observed in the relapsed STUMPs compared to the other samples. Through the combination of genomic data, the association between DNA hypomethylation and genomic instability was confirmed. These findings provide valuable insights into the complex interactions between epigenetic modifications and tumorigenesis in US [81]. There are many challenges in the study of DNA methylation in US, including an inadequate understanding of the underlying mechanisms, limited sample sizes, and methodological imperfections. Recently, WGBS has been successfully applied to the study of other tumors, such as gastric cancer [82, 83], prostate cancer [84], and breast cancer [85], offering hope for overcoming these abovementioned obstacles and advancing our comprehension of epigenetic modifications in US. Compared with DNA methylation arrays, WGBS can detect previously undiscovered CpG islands and has greater potential in identifying DNA methylation sites as biomarkers. Therefore, it is iimportant to conduct more relevant research in the underexplored field of US. DNA methyltransferases (DNMTs) are enzymes responsible for DNA methylation, which is a known factor in cancer cells. They can lead either to hypomethylation or hypermethylation of certain CpG regions in the DNA [86]. DNMTs constitute attractive targets for specific therapies. The FDA has approved “epidrugs” such as 5-azacitidine (5-Aza), 5-aza-2-deoxycytidine (DAC), and the second-generation demethylation agent guadecitabine [87]. 

Additionally, noncoding RNA, which do not encode proteins, play important roles in the oncogenesis of US, influencing gene expression and regulation, cell metabolism, and disease occurrence. Elevated miR-143 and miR-145 expression is a shared feature with leiomyosarcoma and high miR-181b expression which is associated with low expression of its predicted PI3K pathway targets AKT3 and MTOR is more common in uLMS per data obtained by TCGA analysis [14]. Yeheli Ravid et al. conducted a comparative analysis of miRNA profiles between ESS and uLMS, including a comparison of miRNA signatures between primary and metastatic uLMS using miRNA arrays. The differential expression of 94 miRNAs revealed several pathways influenced by miRNAs, including FZD6, which is involved in the Wnt signaling pathway. Notably, FZD6 was found to be significantly overexpressed in metastatic uLMS compared with primary uLMS, suggesting its potential role in regulating cell invasion [88]. In a separate study, researchers conducted a thorough miRNA sequencing analysis on uLMS lesions and uterine leiomyomas. They discovered that the expression level of miR-10b-5p was significantly lower in uLMS than in uterine leiomyoma. Furthermore, the overexpression of miR-10b-5p was demonstrated to suppress the proliferative capacity of sarcoma cells and impede their transition from the G1 phase to the G2/M phase [89]. The mechanisms of epigenetics and corresponding potential therapeutic strategies for uLMS and ESS can be found in the comprehensive review by Bruna Cristine et al. [90]

### Transcriptomics

Transcriptomics, which is downstream of genomics, is used to reveal the rules and mechanisms of gene expression, and their relationships with biological traits and evolutionary relationships. In the realm of transcriptomics, high-throughput methods have emerged as powerful tools for exploring the complex landscape of gene expression. These methods enable researchers to comprehensively analyze the transcriptome, the collection of all RNA molecules within a cell or organism, at an unprecedented scale and resolution.

Using gene expression data from TCGA, Yang An et al. delineated two distinct molecular subtypes of uLMS. Subtype I, characterized as low-grade uLMS, exhibited the overexpression of genes associated with smooth muscle function, including LMOD1, SLMAP, MYLK, and MYH11. Conversely, subtype II tumors, designated high-grade uLMS, presented a greater tumor weight and invasion rate, along with the overexpression of genes implicated in epithelial-to-mesenchymal transition and tumorigenesis pathways, such as CDK6, MAPK13, and HOXA1. Notably, their study also revealed that these distinct molecular subtypes exhibit varying responses to chemotherapy [91]. 

Researchers at Memorial Sloan Kettering Cancer Center (MSKCC) further categorized uLMS into two distinct molecular subtypes and delved into their clinical characteristics. These findings revealed significant differences in prognosis between these two subtypes [92]. By comparing gene expression data with those of other benign or malignant uterine tumors, US can be used to make a more precise diagnosis. Crystal L. Adams et al. identified five genes that are overexpressed in uLMS, namely CHI3L1, MELK, PRC1, TOP2A, and TPX2, whereas uterine leiomyoma exhibited the overexpression of two genes, HPGD and TES. This analysis was conducted by examining the gene expression profiles of uLMS, uterine leiomyoma, and normal myometrial tissues. The discovery of this unique gene expression signature offers the potential to accurately differentiate these tumor types in their early stages, providing prognostic factors and novel therapeutic targets for the treatment of uLMS [93]. RNA-seq has revealed specific gene mutations that delineate intriguing subtypes of US. Abbas Agaimy et al. described 13 uterine stromal neoplasms harboring KAT6B::KANSL1 (*n* = 11) and KAT6A::KANSL1 (*n* = 2) fusion genes. Initially, these neoplasms were diagnosed primarily as LG-ESS. While these tumors exhibit variable overlap with LG-ESS, they typically present as well-circumscribed masses, lacking the extensive permeative and angioinvasive growth patterns characteristic of LG-ESS. The relationship between KAT6B/A::KANSL1 fusion-positive uterine stromal neoplasms and LG-ESS remains unclear [94]. Similarly, Brunetti M. et al. described a novel fusion gene, GREB1-NCOA2, in US via RNA-seq. This chimeric transcript represents an in-frame fusion between exon 3 of GREB1 and exon 15 of NCOA2, marking the first report of such a finding [95]. Furthermore, regarding the GREB1 gene, Cheng-Han Lee et al. utilized RNA-seq and other methodologies to identify four USs harboring rearranged GREB1, a crucial factor in the sex hormone pathway. These rearrangements included GREB1-NCOA2, and the novel fusions of GREB1-NR4A3, GREB1-SS18, and GREB1-NCOA1 [96]. 

Like genomic methods, transcriptomics is used to identify and predict the relationships between gene expression and patient prognosis or treatment. Jian-Guo Zhou et al. accessed US data, including RNA-seq expression profiles and clinical information from TCGA. Utilizing six genes—FGF23, TLX2, TIFAB, RNF223, HIST1H3A, and AADACL4—they developed a prognostic signature designed to predict risk levels. This signature effectively divides USs into two distinct groups: low-risk and high-risk [97]. 

Recently, single-cell RNA sequencing (scRNA-seq) has gained significant attention as a high-throughput method for interrogating transcriptional heterogeneity at the single-cell level. This method enables the profiling of individual cells within a complex tissue or organ, revealing the diverse transcriptional states and cell types that contribute to tissue function and disease pathogenesis. With the significant advancements in sequencing depth and resolution, scRNA-seq has emerged as a formidable tool for tumor research. This method has ushered in a new era of omics by enabling the examination of tumor tissue heterogeneity with “single cell-sized” precision. Despite its potential, scRNA-seq has yet to be explored in the context of US. Similarly, spatial transcriptomics—another approach for studying genetic heterogeneity across different regions of tumor tissue—has also not been investigated in US.

### Proteomics and Metabolomics

In the realm of proteomics, high-throughput methods have played a pivotal role in the comprehensive analysis of protein expression and function. These methods enable researchers to rapidly identify, quantify, and characterize proteins within complex biological systems, providing insights into cellular processes, disease mechanisms, and potential therapeutic targets (Table 4). One of the most widely used high-throughput methods in proteomics is mass spectrometry (MS). MS allows for the precise measurement of the mass and abundance of proteins, enabling the identification and quantitation of thousands of proteins in a single experiment. Liquid chromatography-mass spectrometry (LC-MS) and gel-based proteomics, such as two-dimensional gel electrophoresis, have become standard approaches for protein profiling.

Jessica Burns et al. constructed a proteomic landscape of soft tissue sarcomas, including nine USs. Their analysis revealed the existence of three distinct molecular subtypes—immune cold, classical, and dedifferentiated—each with unique biological characteristics and survival outcomes [98]. By comparing protein expression levels in tumor tissue with those in normal tissue, it is possible to identify potential tumor-specific biomarkers. These biomarkers can play crucial roles in screening and early diagnosis, thereby enhancing the overall management and treatment of US [99]. Through proteomics analysis of uterine leiomyoma and uLMS, major vault protein(MVP) immunohistochemistry was found to have a sensitivity of 50% and specificity of 100% in the comparison of uterine leiomyoma and uLMS [100]. Given the inherent resistance to chemotherapy exhibited by US, proteomics has been increasingly employed in efforts to address this challenge. This particular study revealed distinct protein expression patterns between doxorubicin-resistant sarcoma cells and their normal counterparts at the proteome level. Notably, this finding established the crucial role of the RCN1 protein in conferring doxorubicin resistance and mitigating doxorubicin-induced DNA double-strand breaks [101]. Currently, targeted drugs play a significant role in tumor treatment. Proteomics plays a pivotal role in screening tumor targets for precision therapy. A study conducted by Ruriko Nakae et al. employed iTRAQ-based quantitative proteomic analysis to identify CD70 overexpression in uLMS. These findings suggest that CD70 may serve as a potential therapeutic target for uLMS [102]. 

Metabolomics also holds its own place in the field of postgenomics in the context of tumors. The research methods used in metabolomics include primarily untargeted metabolomics, targeted metabolomics, and imaging mass spectrometry (IMS). Metabolites are distributed in the blood, urine, cerebrospinal fluid, and other bodily fluids. The detection of metabolites in bodily fluids through high-throughput methods has been widely used to screen for tumor markers in various cancers. Changes in plasma metabolites in the pancreas, the body’s largest endocrine organ, can be used to predict the prognosis of late-stage pancreatic cancer [103]. Similarly, some researchers have used untargeted metabolomics to analyze serum and fecal samples from liver cancer patients, revealing the role of metabolites in liver cancer [104]. However, the difficulty in the preoperative diagnosis of US makes it particularly challenging to obtain patient samples prospectively, and research in the field of metabolomics for US is still in its early stages.

**Table 4:** Findings about US by Proteomics and Metabolomics

Omics	Detection Methods	Result	Ref
Proteomics	MS	Expression of MVP can be used to distinguish ULM and ULMS	[100]
Proteomics	2D-DIGE	RCN1 is a useful diagnostic marker and therapeutic candidate	[101]
Proteomics	MS	CD70 may serve as a potential therapeutic target for uLMS	[102]
Proteomics	LC/MS	Three proteomics subtypes with distinct myogenesis and immune features anatomical site distribution and survival outcomes were identified	[98]
Metabolomics	HILIC-HRMS	Expression of glutamine metabolic genes such as SLC1A5, GLS, GOT1 and PPAT1 increase after silence E2F1 expression	[105]

*MS* mass spectrometry, *2D-DIGE* Two-dimensional fluorescence difference in gel electrophoresis, *LC/MS* liquid chromatography/mass spectrometry, *HILIC-HRMS* Hydrophilic Interaction Liquid Chromatography coupled to high resolution mass spectrometry

## Multi-omics in Uterine Sarcoma

**Fig. 3:**
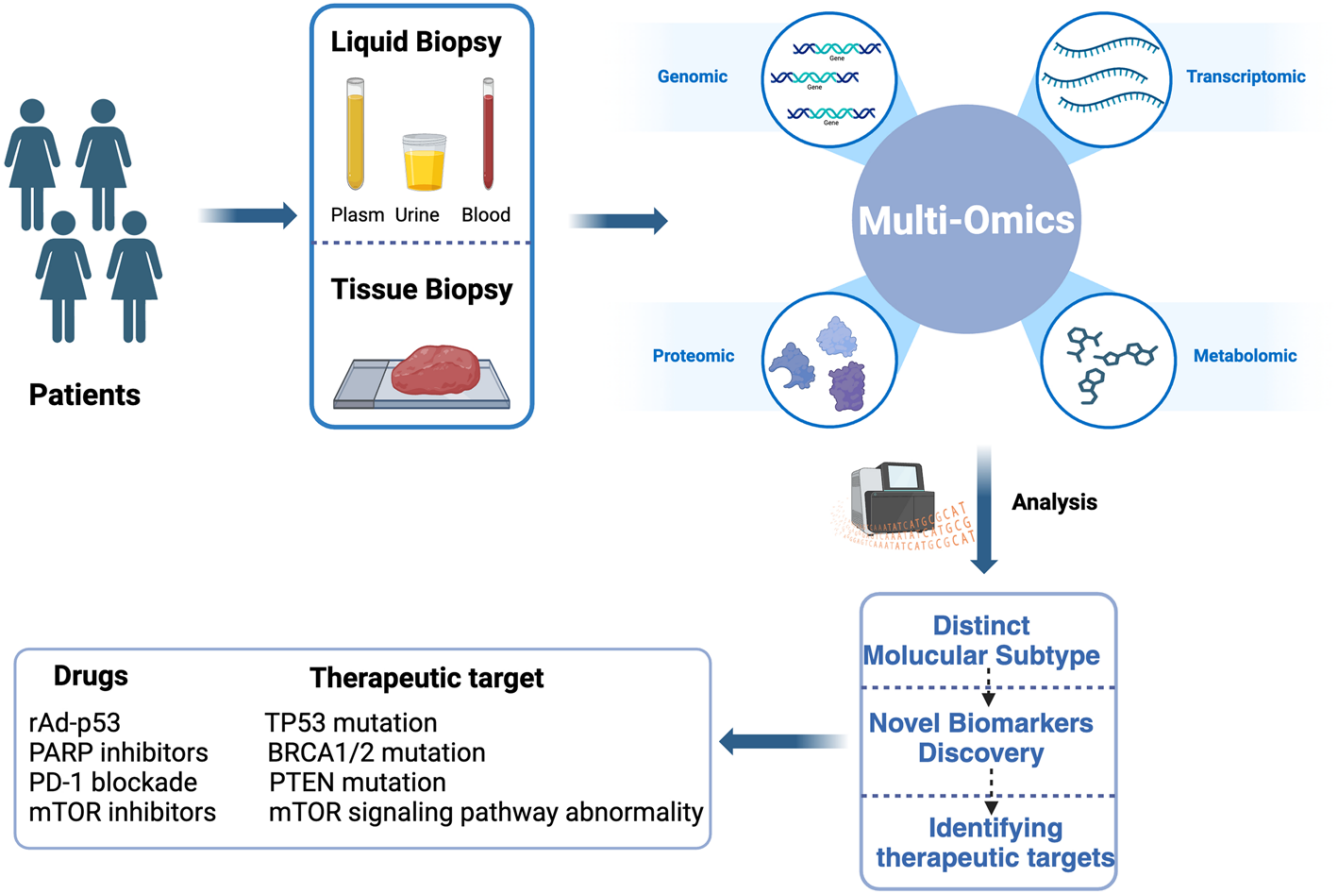
The research process and some findings of multi-omics studies in uterine sarcoma. By collecting surgical tissue or tumor samples from peripheral blood of uterine sarcoma patients, multi-omics sequencing such as genomics, transcriptomics, proteomics, and metabolomics is performed to conduct molecular typing, biomarker screening, and identification of potential therapeutic targets. (Created with BioRender.com)

With the wide application of high-throughput technologies, researchers are now capable of generating vast omics datasets across various molecular levels, including genome, transcriptome, proteome, epigenome, and metabolome. By integrating multi-omics data analysis, we can gain a deeper understanding of the intricate molecular mechanisms and genetic foundations underlying complex disease traits. Moreover, on the basis of these molecular characteristics and pathogenesis, corresponding drugs or other treatment methods can be selected to inhibit tumor progression (Fig. 3).

### Multi-omics Perspective on the analysis of uterine sarcomas

TCGA established a sarcoma cohort (TCGA-SARC) that included uLMS and utilized a multi-omics approach to investigate the pathogenesis and progression of the disease. This innovative study explored sarcomas from various perpectives, including genomics, transcriptomics, epigenomics, and proteomics, offering a comprehensive insight into these intricate malignancies [14]. Using TCGA data on uLMS, Jungmin Choi et al. conducted a comprehensive analysis employing WGS, WES, and RNA-seq. Meticulous examination revealed recurrent somatic mutations in genes such as TP53, MED12, and PTEN. Additionally, they discovered CNVs including amplifications in regions such as 5p15.33 (TERT), 8q24.21 (C-MYC), and 17p11.2 (MYOCD, MAP2K4). Fusion events involving tumor suppressors or oncogenes have also been detected, with many disrupting RB1, TP53, and ATRX/DAXX. Notably, one fusion (ACTG2-ALK) exhibited potential targeting activity. Furthermore, defects in homologous recombination DNA repair (HRD, observed in 25% of cases) and microsatellite instability (MSI, present in 2%) were identified in uLMS, providing valuable insights into the molecular mechanisms underlying this malignancy [106]. Ju-Yoon Yoon et al. conducted a comprehensive study using WES and RNA-seq on two cases of myxoid STUMPs and seven cases of myxoid leiomyosarcoma, with the aim of elucidating the molecular characteristics of this rare US subtype. Their findings revealed that myxoid STUMPs lacked prevalent driver mutations and fusions but frequently presented significant copy number variant burdens, reflected in elevated genomic indexes. Notably, they did not observe BCOR structural aberrations, PLAG1 fusions, or other sarcoma gene fusions in these tumors. These insights provide valuable clues into the molecular mechanisms underlying myxoid STUMPs and uLMS, contributing to a deeper understanding of this rare malignancy [107]. 

Multi-omics analysis offers a multifaceted approach to examining genomic alterations in US, with the ultimate goal of differentiating or defining distinct molecular subtypes of these tumors. In a recent study, Alexis et al. combined CGH analysis with whole RNA-seq in US to gain a comprehensive understanding of the disease. These findings revealed that KAT6B::KANSL1 fusion occurs consistently between exons 3 of KAT6B and 11 of KANSL1. Through clustering analysis, they identified a novel subtype of US, suggesting that this particular subtype harboring the KAT6B::KANSL1 fusion may represent a unique clinicopathologic entity. Although closely related, this subtype appears to be distinct from LG-ESS, providing valuable insights into the molecular heterogeneity of US and potentially leading to more tailored treatment strategies [27]. Similarly, Pier Selenica et al. conducted DNA and RNA sequencing to identify several genetic alterations, including mutations in TP53, TSC2, RB1, ATRX, MED12, and BRCA2, as well as amplifications of CDKN2A, FGFR3, NTRK1, and ERBB3. Furthermore, gene rearrangements such as those in JAZF1-SUZ12, DNAJB6-PLAG1, and SFPQ-TFE3 were detected in three tumors, potentially indicating novel subtypes. These biomarker discoveries enabled the diagnosis of patients with previously unrecognized types of the disease. This comprehensive genomic analysis provides valuable insights into the molecular heterogeneity of tumors and may inform more personalized treatment strategies [108]. Additionally, based on DNA and RNA sequencing performed in US with BCOR gene rearrangement, another study revealed that BCOR rearrangement in US may be sensitive to CDK4/6 inhibition [109]. 

Furthermore, gene expression profiling, coupled with upstream and downstream sequencing data, can facilitate the identification of potential biomarkers for US. Raul Maia Falcão et al. aimed to elucidate the role of PSMB9 in the tumorigenesis of uLMS and patient outcomes by analyzing transcriptomics and proteomics data derived from uLMS, uterine leiomyomas, and normal adjacent myometrium. These findings revealed that the immunoproteasome pathway is upregulated in uLMS, and that PSMB9 exhibits heterogeneous expression patterns. Notably, a high CD8+/PSMB9 ratio was associated with improved overall survival. These insights provide valuable information for understanding the molecular mechanisms underlying uLMS and may lead to the development of more effective therapeutic strategies [110]. In another study, researchers combined proteomics and metabolomics methods to investigate soft tissue sarcomas. They successfully developed a predictive score that accurately forecasts 2-year survival rates, achieving a sensitivity of 84.4% and a specificity of 84.6%. This score is determined by the combined analysis of acetate, triglycerides, low-density lipoprotein-2 (LDL-2), and red blood cell count, all of which have the potential to serve as prognostic biomarkers. This comprehensive approach offers valuable insights into the biological processes underlying soft tissue sarcomas and may lead to improved patient outcomes through more personalized treatment strategies [111]. 

### Artificial intelligence applied in US combined with multi-omics

**Fig. 4:**
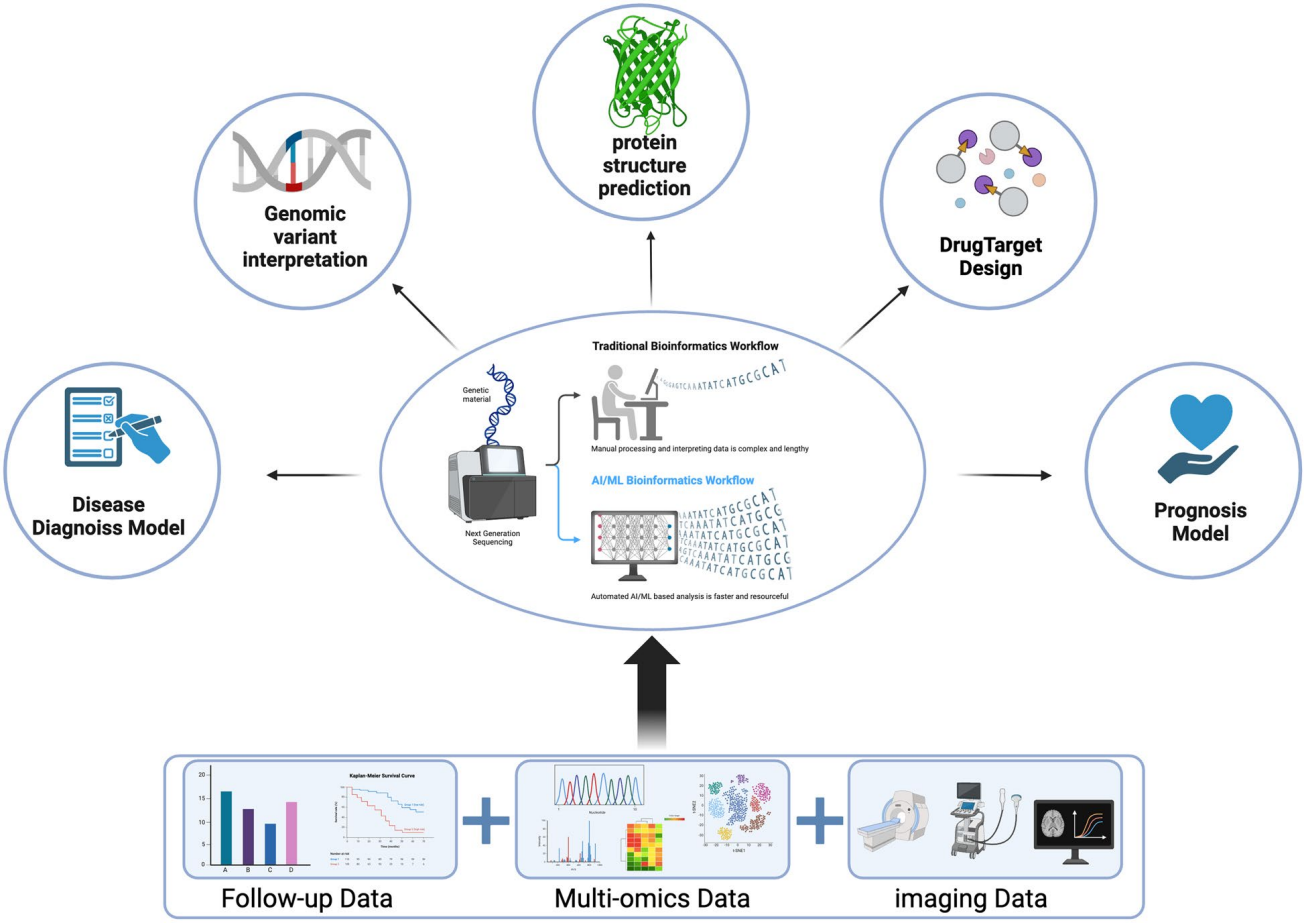
The application of artificial intelligence in integrating multi-dimensional data for uterine sarcoma. Compared to traditional manual analysis, the artificial intelligence-based bioinformatics workflow improves efficiency and accuracy by analyzing large amounts of data. It combines clinical data with omics data to conduct analyses such as genome variant interpretation, protein structure prediction, disease diagnosis, and drug target design. (Created with BioRender.com)

 In recent years, significant advancements in computer science have led to remarkable breakthroughs in various biomedical fields, facilitated by the emergence of artificial intelligence (AI). This technology has revolutionized areas such as genomic variant interpretation, protein structure prediction, disease diagnosis, and drug discovery, demonstrating its potential to revolutionize the biomedical landscape [112]. (Fig. 4)

AI encompasses a vast array of concepts, with machine learning (ML) serving as a crucial component. The advent of genome sequencing and the widespread adoption of deep sequencing methods have significantly influenced all omics disciplines, leading to a growing need for multi-omics analysis and data interpretation leveraging AI methods. These trends are now dominant, highlighting the importance of AI in biomedical research and applications [113]. By integrating machine learning with multi-omics databases, we can potentially identify crucial genes or gene pathways that play significant roles in biological processes. In a study conducted by Ke Y et al., the DPP6 and MFAP5 genes were identified through the application of the least absolute shrinkage and selection operator (LASSO) and support vector machine recursive feature elimination (SVM-RFE) methods, which are widely used in pattern recognition and machine learning. These findings suggest that these genes may influence the occurrence of uLMS through immune-related pathways, providing valuable insights into the molecular mechanisms underlying this disease [114]. Machado-Lopez et al. developed a machine learning approach that identifies gene expression patterns to differentiate between diseases. This model, which relies on transcriptomic signatures from the research group, comprises 19 differentially expressed genes and successfully classifies all samples in the validation set, demonstrating its effectiveness in disease discrimination [115]. Deep learning (DL) is a research field of ML, and it is widely used in a diagnostic or prognostic mode. X. Yang et al. employed several traditional machine learning (ML) methods alongside cutting-edge deep learning (DL) models to differentiate tissue images as benign or malignant. These findings demonstrate that well-trained learning algorithms have the potential to assist in diagnosing low-grade endometrial stromal sarcoma (LGESS) [116]. Radiomics is a rapidly evolving field of research concerned with the extraction of quantitative metrics—the so-called radiomic features—within medical images [117]. DL and radiomics allow for the discovery of novel patterns in medical images [118]. V. Chiappa et al. utilized radiomics within the TRACE4 radiomic platform on preoperative images from the United States following the guidelines of the International Biomarker Standardization Initiative. The objective of these studies was to develop, validate, and assess a classification model for distinguishing myometrial tumors, ultimately demonstrating promise in the differential diagnosis of mesenchymal tumors [119]. Mahrooz Malek et al. developed a diagnostic algorithm for preoperatively differentiating US from uterine leiomyoma through a supervised ML method using multiparametric MRI [120]. 

With the advancement of disease research, high-throughput sequencing has generated a vast amount of molecular data. Clinicians are now faced with the challenge of how to utilize these data effectively for clinical diagnosis and treatment. AI has successfully addressed the obstacles in integrating various types of clinical data, such as imaging graphics and patient follow-up data, with molecular sequencing data such as gene expression matrices and molecular mutation VCF files. Through model construction and data mining, precision medicine is made possible. As technology continues to advance and applications expand, AI is poised to play an increasingly vital role in the field of medicine.

## Conclusions

Multi-omics have already become essential methods for elucidating the complexity of tumorigenesis and tumor progression, especially when facing US which is a rare tumor. Multi-omics research provides potential diagnostic and therapeutic biomarkers for tumor research. Multi-omics research on uterine sarcomas is relatively scarce, yet it has still made certain progress. Martee L. Hensley et al. conducted exome sequencing in 107 USs and found that uLMS patients carrying BRCA2 mutations are expected to clinically benefit from the use of PARP inhibitors [55]. Suzanne George et al. also highlights the therapeutic potential of anti-PD-1 treatment in patients with PTEN-deficient uLMS by WES and RNA-seq [72]. Although multi-omics have extensively promoted the discovery of potential biomarkers of US, the gap between multi-omics research findings and clinical applications persists. Researchers should clarify research protocols based on sample types, omics technology characteristics, and statistical analysis methods, thereby enhancing the clinical application value of research related to US.

In the field of omics, we have now entered the era of single-cell omics and spatial omics. The core of this technology lies in its ability to capture and analyze subtle differences between single cells, revealing the heterogeneity among cells and their unique roles in biological processes. Spatial omics facilitates the exploration of cell interactions and spatial organization within tissues, shedding light on the intricate relationships between cells and their surrounding microenvironments offering enhanced precision in disease diagnosis and treatment. However, two major issues greatly hampered the application of single-cell and spatial omics technologies in US. Firstly, USs, rare tumors, are difficult to obtain fresh specimens which are usually diagnosed in FFPE sections. Now with the development of sequencing technology, FFPE samples currently become amenable for omics sequencing. Secondly, definitive biomarkers are essential to identify malignant cell populations in single cell and spatial omics. However, these biomarkers of various histological subtypes US have not been figured out.

Considering the restriction of multi-omics, multi-omics assays were unaffordable in the past decades. Nowadays, the cost of multi-omics is decreasing thus the large-scale sequencing cohort is available. Due to the limited sample size in US omics research, researchers attempt to pool the results from multiple studies. However, this approach poses the following challenges: firstly, batch effects; secondly, the difficulty in integrating different types of data, as some studies focus on DNA, others on RNA, and still others on proteins. Multi-omics databases, such as TCGA and cBioPortal created by MSKCC, store billions of data. The application of AI algorithms assists researchers to remove batch effects between different studies. AI algorithms also could conduct a joint analysis by combining research data on DNA, RNA, and proteins.

In the future, the availability of FFPE samples, the reduction in sequencing costs, and the implementation of multicenter clinical trials will drive the application of single-cell omics and spatial omics in US. Furthermore, AI algorithms and other analytical tools will be used to identify new biomarkers of malignant cell populations, thereby being advantageous to the application of single-cell omics and spatial omics in US.

In summary, US is an important health burden for women, while multi-omics profiling has provided new insights into US preoperative diagnosis and individual therapy. Although many challenges remain, there are substantial ongoing efforts to address these issues and promote the clinical translation of multi-omics findings on US. These technologies can reveal the molecular profile of US, thereby discovering biomarkers and therapeutic targets, which are crucial in developing personalized treatment strategies.

## Data Availability

No datasets were generated or analysed during the current study.

## Abbreviations

US: Uterine Sarcoma
ULM: Uterine Leiomyoma
DWI: Diffusion Weighted Imaging
NGS: Next-Generation Sequencing
FIGO: the international Federation of Gynecology and Obstetrics
uLMS: Uterine Leiomyosarcoma
ESS: Endometrial Stromal Sarcoma
UUS: Undifferentiated Uterine Sarcoma
AS: Adenosarcoma
NCCN: the National Comprehensive Cancer Network
MF/HPF: Mitose/high-power fields
SMA: Smooth Muscle Actin
EMA: Epithelial Membrane Antigen
TCGA: The Cancer Genome Atlas
CNV: Copy Number Variation
TP53: Tumor Protein 53
RB1: RB Transcriptional corepressor 1
ATRX: ATRX chromatin remodeler
PDCD1: Programmed Cell Death 1
MED12: Mediator Complex Subunit 12
PGR: Progesterone Receptor
PLAG1: PLAG1 Like Zinc Finger 1
WHO: World Health Organization
LG-ESS: Low-Grade Endometrial Stromal Sarcoma
HG-ESS: High-Grade Endometrial Stromal Sarcoma
LVSI: Lymphatic Vascular Invasion
BCOR: BCL6 Corepressor
ITD: Internal Tandem Duplication
CGH: Array Comparative Genomic Hybridization
FISH: Fluorescence in Situ Hybridization
IHC: Immunohistochemistry
RACE-PCR: 3V-Rapid Amplification of cDNA Ends PCR
MPS: Massively Parallel Sequencing
RNA-seq: RNA Sequencing
NTRK: Neurotrophic Receptor Tyrosine Kinase
BRG1: S-ribonuclease Binding Protein Family Protein
SMARCA4: SWI/SNF related, matrix associated, actin dependent regulator of chromatin, subfamily a, member 4
MDM2: MDM2 Proto-oncogene
FF: Fresh Frozen
FFPE: Formalin-Fixed Paraffin Embedding
LB: Liquid Biopsy
cfDNA: Circulating Free DNA
ctDNA: Circulating Tumor DNA
lnc-RNA: long non-coding RNA
LMW cfDNA: Low Molecular Weight cfDNA
ULP-WGS: Ultra-low Passage Whole-genome Sequencing
WGS: Whole Genome Sequencing
WES: Whole Exome Sequencing
SNV: Single Nucleotide Variation
TMB: Tumor Mutation Burden
PARP: Poly ADP-Ribose Polymerase
PTEN: Phosphatase and Tensin Homolog
LM-BNs: Leiomyoma with Bizarre Nuclei
FH-LMs: Fumarate Hydratase-Deficient Leiomyomas
ZNF217: Zinc Finger Protein 217
NRDC: Nardilysin Convertase
STUMPs: Smooth Muscle Tumors of Uncertain Malignant Potential
HRR: Homologous Recombination Repair
PFS: Progression-free Survival
MTOR: Mechanistic Target of Rapamycin Kinase
CDKN2A: Cyclin Dependent Kinase Inhibitor 2A
WGBS: Whole-Genome Bisulfite Sequencing
ChIP-seq: Chromatin Immunoprecipitation Sequencing
UTROSCT: Uterine Tumors Resembling Ovarian Sex Cord Tumors
CGIs: CpG Islands
DNMTs: DNA Methyltransferases
FDA: Food and Drug Administration
5-Aza: 5-azacitidine
DAC: 5-aza-2-deoxycytidine
FZD6: Frizzled Class Receptor 6
DEGs: Differential Expressed Genes
scRNA-seq: Single-Cell RNA Sequencing
MSKCC: Memorial Sloan Kettering Cancer Center
MS: Mass Spectrometry
LC-MS: Liquid Chromatography-Mass Spectrometry
MVP: Major Vault Protein
RCN1: Reticulocalbin 1
IMS: Imaging Mass Spectrometry
HRD: Homologous Recombination DNA Repair
MSI: Microsatellite Instability
PSMB9: Proteasome 20S Subunit Beta 9
LDL-2: Low-Density Lipoprotein-2
AI: Artificial Intelligence
ML: Machine Learning
LASSO: Least Absolute Shrinkage and Selection Operator
SVM-RFE: Support Vector Machine Recursive Feature Elimination
DL: Deep Learning
VCF: Variant Call Format
